# Progress in Gene Therapy for Prostate Cancer

**DOI:** 10.3389/fonc.2012.00172

**Published:** 2012-11-19

**Authors:** Kamran A. Ahmed, Brian J. Davis, Torrence M. Wilson, Gregory A. Wiseman, Mark J. Federspiel, John C. Morris

**Affiliations:** ^1^Department of Radiation Oncology, Mayo ClinicRochester, MN, USA; ^2^Department of Urology, Mayo ClinicRochester, MN, USA; ^3^Division of Nuclear Medicine, Mayo ClinicRochester, MN, USA; ^4^Department of Molecular Medicine, Mayo ClinicRochester, MN, USA; ^5^Division of Endocrinology, Mayo ClinicRochester, MN, USA

**Keywords:** gene therapy, sodium-iodide symporter, prostate cancer

## Abstract

Gene therapy has held promise to correct various disease processes. Prostate cancer represents the second leading cause of cancer death in American men. A number of clinical trials involving gene therapy for the treatment of prostate cancer have been reported. The ability to efficiently transduce tumors with effective levels of therapeutic genes has been identified as a fundamental barrier to effective cancer gene therapy. The approach utilizing gene therapy in prostate cancer patients at our institution attempts to address this deficiency. The sodium-iodide symporter (NIS) is responsible for the ability of the thyroid gland to transport and concentrate iodide. The characteristics of the NIS gene suggest that it could represent an ideal therapeutic gene for cancer therapy. Published results from Mayo Clinic researchers have indicated several important successes with the use of the NIS gene and prostate gene therapy. Studies have demonstrated that transfer of the human NIS gene into prostate cancer using adenovirus vectors *in vitro* and *in vivo* results in efficient uptake of radioactive iodine and significant tumor growth delay with prolongation of survival. Preclinical successes have culminated in the opening of a phase I trial for patients with advanced prostate disease which is currently accruing patients. Further study will reveal the clinical promise of NIS gene therapy in the treatment of prostate as well as other malignancies.

## Gene Therapy

Gene therapy was first conceptualized in 1972 with the promise to correct numerous inheritable diseases. Gene therapy functions by using DNA packaged into a vector to insert itself into a human genome and alter the cell in a manner designed to preserve the overall health and life of the organism (Friedmann and Roblin, 1972). By encoding a therapeutic protein, a host of diseases could potentially be treated. Many of the first potential targets for gene therapy were considered to be hematopoietic disorders in which the mechanism of disease was well understood. In these disorders, cells could often be easily manipulated in the laboratory setting (Anderson, 1984). Unfortunately, many of the early promises of gene therapy were dampened by technical challenges involved with gene transfer in high numbers of hematopoietic stem cells and high levels of β-globin gene expression in erythrocytes. Attention then turned to the promise of gene therapy being used in the treatment of severe combined immunodeficiency disorder (SCID). The use of gene therapy with SCID was filled with both successes and failures until one of the major breakthroughs reported in 2000 by Cavazzana-Calvo et al. (2000). This group demonstrated immune reconstitution in five infants with X-linked SCID who underwent gene therapy. This was then followed by Aiuti et al. (2002) describing initial signs of immune reconstitution in two infants with SCID due to adenosine deaminase deficiency (ADA).

Since that time other successes have been reported with the use of gene therapy in adrenoleukodystrophy (Cartier and Aubourg, 2010), Parkinson’s disease (LeWitt et al., 2011), and the retinal disease, Leber’s Congenital Amaurosis (Bainbridge et al., 2008; Cideciyan et al., 2009). These triumphs have renewed the promise of gene therapy in the fight against various disease processes. However, there has been limited success in the use of gene therapy to combat cancer. Although gene therapy approaches have been developed for numerous malignancies, clinical trials completed to date have fallen short of expectations (Humphreys et al., 1999; Hung et al., 2000; Cascallo et al., 2003; Miura et al., 2005; Young et al., 2006). However, one of several areas of progress includes the use of viruses capable of replicating selectively in cancer cells. These oncolytic viruses provide an opportunity to attack a number of mechanistically distinct cellular pathways while also generating progeny viruses capable of spreading throughout a tumor mass (Chiocca, 2002; Ries and Brandts, 2004). In China, gene therapy has been licensed as a regular treatment for head and neck cancer, using an adenovirus (ADV) to deliver the p53 tumor suppressor gene by direct injection into the tumor given in combination with radiotherapy (Peng, 2005).

## Gene Therapy in Prostate Cancer

Prostate cancer represents the second leading cause of cancer death in American men (Ruijter et al., 1999). The majority of men present with curable and localized disease, yet approximately 30% of patients will progress to advanced stages within 10–15 years of diagnosis. Metastatic prostate cancer may have a long course, but it is almost invariably incurable. Typically, systemic hormonal options, such as chronic androgen deprivation therapy (ADT), are initiated when metastatic disease is diagnosed. ADT provides cancer control and palliation for variable periods of time ranging from a few months to several years. For men who progress to the hormone refractory state, treatment with docetaxel chemotherapy is an option (Tannock et al., 2004). At the present time, there is no standard treatment for patients with progressive disease following docetaxel chemotherapy. Metastatic, androgen resistant prostate cancer remains a leading cause of death in American men. For this reason, novel strategies, including those involving gene therapy, are needed.

A number of clinical trials involving gene therapy for the treatment of prostate cancer are ongoing and a few have been completed. These trials are summarized in Table 1. In several clinical trials ADV was injected directly into the prostate. These studies have utilized several transgenes, including p53, Herpes simplex tk, IL-2, IL-12, and RVTP1. In the first reported results of 18 patients with locally recurrent prostate cancer, a single dose, dose escalation study was conducted using an ADV vector with the HSV thymidine kinase gene (Adv/HSV-tk) injected transrectally into one lobe of the prostate followed by ganciclovir (GCV; Herman et al., 1999). There was no local toxicity; the most common adverse events reported were liver transaminase elevations (three Grade 1 and one Grade 4), fever, thrombocytopenia, and leukopenia. In another multiple-dose, dose escalation study, RPR/ING/N201, a replication-defective ADV with a wild type p53 was injected intraprostatically in 17 patients with locally advanced disease every 2 weeks for a total of up to three treatments prior to radical prostatectomy (RP; Sweeney and Pisters, 2000). Only mild toxicities were reported.

**Table 1:** **Prostate cancer gene therapy trials**.

Strategy	Investigator	Date	Institution
Cytokine (GM-CSF) immunotherapy (*ex vivo*)	Simmons	Aug-94	Johns Hopkins
Cytokine (GM-CSF) allogeneic immunotherapy (*ex vivo*)	Simmons	Sep-97	Johns Hopkins
Retrovirus antisense c-myc	Steiner	Sep-95	Vanderbilt
Intradermal vaccinia-PSA	Chen	Sep-95	Naval Medical Academy
Adenovirus ganciclovir/TK	Scardino	Jan-96	Baylor
Intradermal vaccinia-PSA	Kufe	Sep-96	Dana Farber
Intradermal vaccinia-PSA	Sanda	May-97	University of Michigan
Liposome IL-2 Belldegrun	Belldegrun	May-97	UCLA
Adenovirus ganciclovir/TK	Hall	Feb-98	Mt. Sinai Hospital
Adenovirus p53 Belldegrun	Belldegrun	Sep-97	UCLA
Adenovirus p53 Logothetis	Logothetis	Nov-97	MD Anderson
Adenovirus ganciclovir/TK	Kadmon	Feb-98	Baylor
Intramuscular vaccinia MUC-1-IL-2	Figlin	May-98	UCLA
Adenovirus, prostate oncolytic	Simons	Jun-98	Johns Hopkins
Adenovirus, IL-12	Miles	Jun-98	Baylor
Adenovirus, IL-12	Belldegrun	Nov-00	UCLA
Adenovirus, IL-12	Hall	Apr-05	Mt. Sinai Hospital
Adenovirus RVTP1	Kadmon	Aug-06	Baylor
Replicating Ad + EBRT	Movasis	Dec-07	Henry Ford Hospital
Adenovirus, valacyclovir	Shirakawa	Dec-07	Kobe University, Japan
Adenovirus (NTR)	Patel	Jul-09	CR UK Institute for Cancer Studies
Adenovirus (NTR), prodrug CB1954	Onion	Nov-09	CR UK Institute for Cancer Studies
Replicating Ad (hNIS)	Barton	Jul-11	Henry Ford Hospital
Adenovirus (GLIPR1)	Sonpavde	Nov-11	Baylor

*EBRT, External beam radiotherapy; GLIPR1, Glioma pathogenesis-related protein 1; GM-CSF, Granulocyte macrophage colony stimulating factor; hNIS, Human iodide symporter; IL, Interleukin; NTR, Nitro reductase; PSA, Prostate-specific antigen; TK, Thymidine kinase*.

CV706 is a prostate-specific antigen (PSA)-selective, replication-competent ADV that has been shown to selectively kill human prostate cancer xenografts in preclinical models (Rodriguez et al., 1997). A phase I study for the treatment of patients with locally recurrent prostate cancer after radiation therapy was conducted (DeWeese et al., 2001). Twenty patients in five groups were treated with doses ranging from 1 × 10^11^ to 1 × 10^13^ viral particles (vp) delivered by a transrectal ultrasound-guided transperineal technique using a three-dimensional plan. The primary end point was the determination of treatment-related toxicity. The study found CV706 to be safe and not associated with irreversible grade 3 or 4 toxicity. No grade >1 alterations in liver function tests associated with CV706 administration were reported.

A phase I dose escalation clinical trial conducted at Baylor College of Medicine (Herman et al., 1999) of intraprostatic injection of a replication-deficient ADV containing the herpes simplex virus thymidine kinase gene (HSV- tk), followed by intravenous administration of the prodrug GCV, was conducted. One patient at the highest dose level developed an objective response, one each at the three highest dose levels, documented by a fall in serum PSA levels by 50% or more, sustained for 6 weeks to 1 year. This study is reported to be the first to demonstrate anticancer activity of gene therapy in patients with prostate cancer.

A phase I study of replication-competent ADV-mediated gene therapy for locally recurrent prostate cancer involving delivery of two suicide genes, cytosine deaminase and HSV-tk, was the first study reporting injection of replicating ADV in humans (Freytag et al., 2002). Sixteen patients were studied with a maximum viral dose of 10^12^ vp. No dose limiting toxicities were observed and the maximum tolerated dose (MTD) was not defined. A total of 7 of 16 patients demonstrated >25% fall in PSA and two patients are negative for prostate cancer as determined by prostate biopsy after 1 year. This same vector was used in two additional trials combining gene therapy with chemotherapy and radiation, both demonstrating low toxicities, no dose limiting toxicity (DLT) and declines in PSA levels, although the responses were short term in both trials (Freytag et al., 2003, 2007).

A phase I/II clinical trial from the United Kingdom by Patel et al. (2009) reported on direct intraprostatic injection of a defective ADV vector (CTL102) encoding bacterial nitroreductase (NTR) in conjunction with a systemic prodrug. The cohort of patients were split into two groups; one group scheduled for RP received the virus alone, in a dose escalation study to establish tolerability, safety, and expression of NTR. To establish safety and tolerability, a second group of 19 patients with local failure following primary treatment received virus plus prodrug. A total of 14 of these patients had a repeat treatment. Results indicated minimal toxicity and preliminary evidence of a change in PSA kinetics.

Recently, Sonpavde et al. (2011) conducted a phase I clinical trial to evaluate the safety and activity of the neoadjuvant intraprostatic injection of ADV expressing the tumor suppressor gene GLIPR1 for intermediate or high-risk prostate cancer before RP. Nineteen patients received a single injection into the prostate followed 4 weeks later by RP using six vp dose levels. Toxicities included urinary tract infection (*n* = 3), flu-like syndrome (*n* = 3), fever (*n* = 1), dysuria (*n* = 1), and photophobia (*n* = 1). Results indicated biologic antitumor activity and systemic immune responses.

In spite of these initial encouraging results, an optimal gene therapy strategy in prostate cancer has yet to be determined. Effective approaches to gene therapy depend on the ability to deliver therapeutic genes to target cells. The ability to efficiently transduce tumors with effective levels of therapeutic genes has been identified as a fundamental barrier to effective cancer gene therapy (Herrmann, 1995; Waehler et al., 2007). To address this issue, conditionally replicating viruses, including the ADV, have been constructed and their efficacy has been evaluated (Markert et al., 1993; Bischoff et al., 1996; Russell, 2002). It is suggested from previous gene therapy trials in cancer patients that a multimodality approach utilizing chemotherapy and/or radiotherapy may be required in order to maximize efficacy (Chu et al., 2004). The approach utilizing gene therapy in prostate cancer patients at our institution attempts to address these two deficiencies.

## Prostate Cancer Gene Therapy with the Sodium-Iodide Symporter (NIS)

Radioactive iodine (^131^I) is one of the most effective forms of systemic radiotherapy available today (Mazzaferri and Kloos, 2001). Even in advanced cases, thyroid cancer can be effectively treated by radioactive iodine therapy. Radioiodine has great utility in the management of thyroid cancer because thyroid cells possess the ability to concentrate iodide from extracellular fluid. This iodine trapping activity of thyroidal cells is utilized both in the diagnosis as well as treatment of thyroid cancer. Functioning thyroid cancer metastases can be detected by administering radioiodine in the form of ^123^I and then imaging with a gamma camera. Metastases that are imaged by this technique are then targeted for therapy by administration of therapeutic doses of ^131^I to the patient. The success rate of metastatic thyroid cancer treated with ^131^I is reflected in the low mortality of patients suffering from the disease. This is also true for patients with advanced disease and diffuse pulmonary metastases at initial presentation who can be successfully treated by ^131^I, achieving a 10 year survival rate of over 80% (Mazzaferri and Jhiang, 1994).

**Figure 1:**
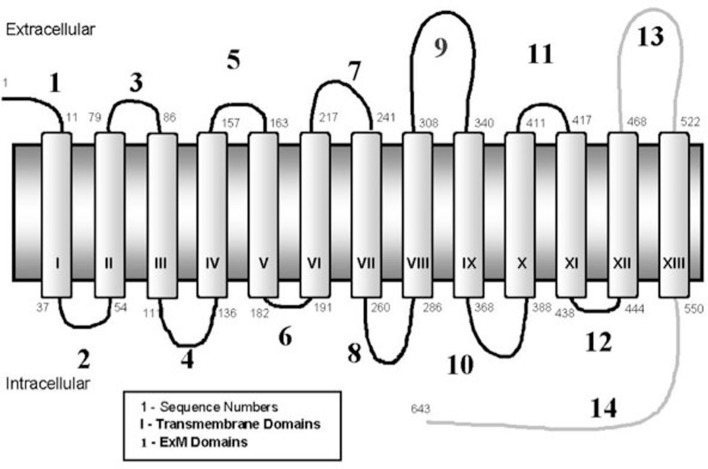
**Structure of the human sodium-iodide symporter (hNIS)**.

The sodium-iodide symporter (NIS) is an intrinsic membrane glycoprotein, with 13 transmembrane domains, which is responsible for the ability of the thyroid gland to transport and concentrate iodide (Figure 1). TSH-regulated NIS expression is specific for thyroid cells, whereas many other organs which lack NIS expression, including the prostate gland, do not concentrate iodide (Spitzweg et al., 1999). Cloning and characterization of the human NIS (hNIS) gene offers the possibility to deliver the gene into non-thyroid cells, thereby allowing those non-thyroidal cells to trap radioiodine. Mayo Clinic researchers have extensively explored the possibility of using this gene as a therapeutic for numerous types of cancers. In addition, other groups have investigated the use of the NIS gene as a therapeutic in breast (Tazebay et al., 2000), brain (Cho et al., 2000), and liver (Haberkorn, 2001) cancers.

**Figure 2:**
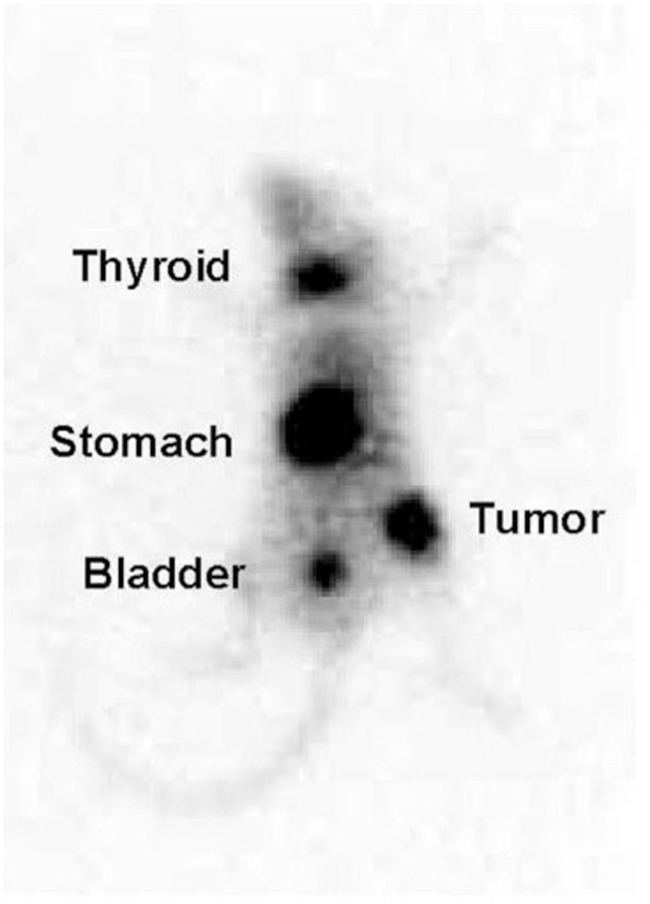
**^123^I scan of a mouse with the adenovirus-derived vector with a non-specific promoter (CMV) carrying the sodium-iodide symporter gene (NIS; Ad-CMV-NIS)**. The infected prostate cancer xenograft over right hip shows radioiodine uptake while there is no uptake over the control tumor on the left hip.

Animal studies have demonstrated that expression of the hNIS gene by plasmid or adenoviral mediated gene transfer in prostate cancer cells results in a large concentration of iodide by the cells (Markert et al., 1993) as visualized in the mouse model depicted in Figure 2. Uptake is of sufficient magnitude and duration *in vivo* that administration of therapeutic doses of ^131^I to animals bearing prostate cancer xenografts results in highly effective therapy of those tumors, achieving complete response rates as high as 80% (Spitzweg et al., 2001).

The characteristics of the NIS gene suggest that it could represent an ideal therapeutic gene for cancer therapy, having several advantages over other investigated genes. Most importantly is its high degree of efficacy. NIS is already a therapeutic gene in that its native expression in thyroid cells is used for therapy of thyroid cancer and hyperthyroidism (Mazzaferri, 1996). It also has a high bystander effect where ^131^I decay, which occurs primarily through emission of β particles that traverse up to 2 mm within the tissue, allows the energy of the decay to be deposited into cells that are neighbors of the cell in which the event occurred. Thus, not every cell must be transfected and express NIS to be effected by radioiodine therapy. This is important for any cancer gene therapy strategy because of the difficulty in achieving 100% transfection with the therapeutic gene. NIS is a normal human gene and protein, which means its expression in cancer cells is unlikely to be toxic or limit its efficacy in patients. Because expression of NIS is not toxic to the cells in the absence of radioiodine, no special techniques to avoid killing of virus producing cells are required for generation of the viral vectors. Thus, production of vectors only utilizes standard techniques. Furthermore, NIS has a high specificity. Although NIS is a normally expressed human gene, its native expression is confined only to a small number of organs, including the thyroid, where its expression is highest. Therefore, the uptake of radioiodine in these organs does not cause significant morbidity outside the intended organ of interest. This strategy protects normal tissues while targeting the site of treatment in the prostate. Lastly, in contrast to other currently used therapeutic genes, NIS expression can be directly monitored and quantified non-invasively using radioiodine scanning and nuclear medicine techniques.

## Progress at the Mayo Clinic

Published results from Mayo Clinic researchers have indicated several important successes with the use of the NIS gene and prostate gene therapy. It has been demonstrated that transfer of the hNIS gene into prostate cancer using ADV vectors *in vitro* and *in vivo* results in highly efficient uptake of radioactive iodine (Spitzweg et al., 1999). This was also shown using a recombinant measles virus derived from the Edmonston vaccine strain (MV-Edm). Intratumoral administration of the virus resulted in statistically significant tumor growth delay (*P* = 0.004) and prolongation of survival (*P* = 0.001) in a subcutaneous xenograft model. Studies have also shown that adenoviral constructs containing NIS used to transfect prostate cancer xenografts demonstrated uptake of radioiodine sufficient to induce tumor volume reduction up to 80% in comparison to control tumors after treatment with a single dose of ^131^I (Spitzweg et al., 2001; Kakinuma et al., 2003). This finding could be particularly useful for smaller tumors or micrometastases.

**Figure 3:**
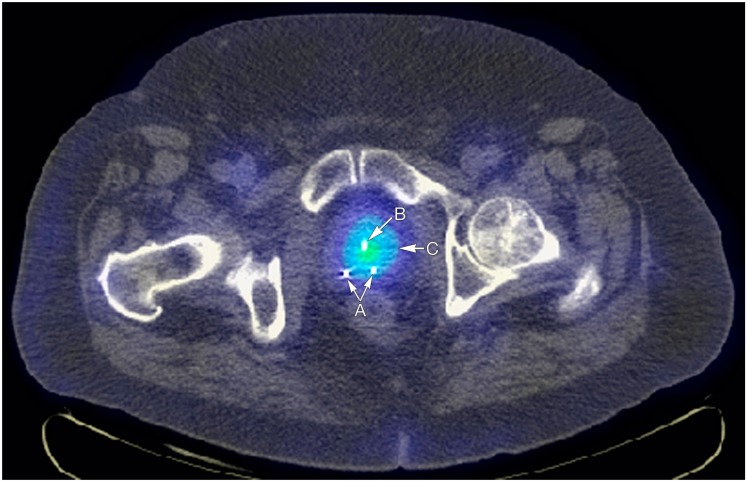
**Arrow A points to fiducial markers for external beam radiotherapy image guidance, arrow B points to Foley catheter, and arrow C to increased uptake of I-123 in the prostate attributable to incorporation of the NIS gene in to prostate cells via *in situ* injections**.

Researchers at Mayo have also completed biotoxicity and biodistribution studies in dogs, which revealed the successful introduction of localized NIS expression in the prostate gland with no vector-related toxicity observed. No animals experienced any surgical complications, and laboratory panels showed no significant change following therapy providing further support for the translation of this work to the clinical setting (Dwyer et al., 2005). Our experience with ADV-mediated NIS transfer and radioiodine therapy has been confirmed in large animal models, which has culminated in the opening of a phase I trial for patients with advanced prostate disease. Patients in the study have all experienced local recurrence following external beam radiation treatment (http://clinicaltrials.gov/ct/show/NCT00788307). The study is currently open and accruing patients. Figure 3 displays the uptake of ^131^I in the prostate following NIS gene transfer.

This dose escalation trial will help determine how much radioiodine can be safely injected to achieve the maximal therapeutic effect. In thyroid cancer, radioiodine is administered in a fixed dose regimen. The 2009 American Thyroid Association guidelines recommend using the minimal activity necessary to achieve successful ablation (between 30 and 100 mCi; Cooper et al., 2009). NIS gene therapy has the potential for a significant therapeutic effect with direct injection of the vector into the prostate which permits concentration of radioiodine directly in the prostate. However, preclinical animal models have demonstrated some uptake of radioiodine in thyroid and gastric mucosa, which may ultimately limit dose escalation and the achievable therapeutic effect in patients (Dwyer et al., 2005). Further clinical study will reveal the maximal dose necessary to achieve a therapeutic effect while limiting toxicity.

## Future Directions

As with many current treatment regimens for cancer, gene therapy utilized as a monotherapy may not be the sole solution. A multimodal approach combining virus based gene therapy with chemotherapy and/or radiotherapy may be necessary for more complete tumor eradication. The development of phase I trials using a multimodal approach to therapy will likely be necessary once a gene therapy strategy has been optimized.

Due to the technical aspects involved in the clinical application of gene therapy, progress has been noted but there is much work left to accomplish. Although the majority of our work to date has utilized the prostate cancer model, researchers have also demonstrated the utility of NIS mediated gene therapy *in vitro* and/or *in vivo* for several other tumor models, including breast, pancreas, colon, medullary thyroid, ovarian carcinomas (Scholz et al., 2005; Dwyer et al., 2006a,b), and in multiple myeloma (Dingli et al., 2004). Initial preclinical results appear promising; however, only further study will reveal the clinical promise of NIS gene therapy in these cancers.

## Conflict of Interest Statement

The authors declare that the research was conducted in the absence of any commercial or financial relationships that could be construed as a potential conflict of interest.
